# Northern Gannet foraging trip length increases with colony size and decreases with latitude

**DOI:** 10.1098/rsos.240708

**Published:** 2024-09-04

**Authors:** Bethany L. Clark, Freydís Vigfúsdóttir, Sarah Wanless, Keith C. Hamer, Thomas W. Bodey, Stuart Bearhop, Ashley Bennison, Jez Blackburn, Sam L. Cox, Kyle J. N. d’Entremont, Stefan Garthe, David Grémillet, Mark Jessopp, Jude Lane, Amélie Lescroël, William A. Montevecchi, David J. Pascall, Pascal Provost, Ewan D. Wakefield, Victoria Warwick‐Evans, Saskia Wischnewski, Lucy J. Wright, Stephen C. Votier

**Affiliations:** ^1^ BirdLife International, The David Attenborough Building, Pembroke Street, Cambridge CB2 3QZ, UK; ^2^ University of Exeter, Penryn TR10 9FE, UK; ^3^ Department of Sustainability, Ministry of Food, Agriculture and Fisheries, Borgartún 26, 105, Reykjavik, Iceland; ^4^ Institute for Sustainability Studies, University of Iceland, Gimli building, Sæmundargata, 105, Reykjavik, Iceland; ^5^ UK Centre for Ecology & Hydrology, Penicuik EH26 0QB, UK; ^6^ School of Biology, University of Leeds, Leeds LS2 9JT, UK; ^7^ School of Biological Sciences, University of Aberdeen, Aberdeen AB24 3FX, UK; ^8^ British Antarctic Survey, Madingley, Cambridge CB3 0ET, UK; ^9^ British Trust for Ornithology, The Nunnery, Thetford, Norfolk IP24 2PU, UK; ^10^ School of Biological, Earth & Environmental Sciences, University College Cork, Cork T23 N73K, Ireland; ^11^ MaREI Centre, Environmental Research Institute, University College Cork, Cork P43 C573, Ireland; ^12^ Psychology Department, Memorial University of Newfoundland, St John’s, Newfoundland, Newfoundland and Labrador A1C 5S7, Canada; ^13^ Research and Technology Centre (FTZ), University of Kiel, Büsum, Germany; ^14^ CEFE, University of Montpellier, CNRS, EPHE, IRD, Montpellier, France; ^15^ RSPB Centre for Conservation Science, Sandy, Bedfordshire SG19 2DL, UK; ^16^ Point Blue Conservation Science, Petaluma, CA 94954, USA; ^17^ MRC Biostatistics Unit, University of Cambridge, Cambridge CB2 0SR, UK; ^18^ Ligue pour la Protection des Oiseaux, Réserve Naturelle Nationale des Sept-Iles, Pleumeur Bodou 22560, France; ^19^ Department of Geography, Durham University, Lower Mountjoy, South Road, Durham DH1 3LE, UK; ^20^ Lyell Centre, Institute for Life and Earth Sciences, Heriot-Watt University, Edinburgh EH14 4AS, UK

**Keywords:** central place foraging, coloniality, species distributions, bio-logging, predator–prey, seabird

## Abstract

Density-dependent competition for food influences the foraging behaviour and demography of colonial animals, but how this influence varies across a species’ latitudinal range is poorly understood. Here we used satellite tracking from 21 Northern Gannet *Morus bassanus* colonies (39% of colonies worldwide, supporting 73% of the global population) during chick-rearing to test how foraging trip characteristics (distance and duration) covary with colony size (138–60 953 breeding pairs) and latitude across 89% of their latitudinal range (46.81–71.23° N). Tracking data for 1118 individuals showed that foraging trip duration and maximum distance both increased with square-root colony size. Foraging effort also varied between years for the same colony, consistent with a link to environmental variability. Trip duration and maximum distance also decreased with latitude, after controlling for colony size. Our results are consistent with density-dependent reduction in prey availability influencing colony size and reveal reduced competition at the poleward range margin. This provides a mechanism for rapid population growth at northern colonies and, therefore, a poleward shift in response to environmental change. Further work is required to understand when and how colonial animals deplete nearby prey, along with the positive and negative effects of social foraging behaviour.

## 1. Background

Foraging is strongly affected by group living; there can be benefits via social information transfer [1,2] and costs via intraspecific competition for food [3–5]. Most studies of colonial animals suggest that foraging effort covaries with population size because large colonies deplete nearby prey and so individuals must travel further to forage [6–9]. This relationship may be altered by local environmental conditions that impact food availability and thus affect foraging behaviour [10,11], including at species’ range margins where competition may be lower [12]. Nevertheless, multi-population studies tend to be based on a small number of colonies and confined to a relatively small proportion of a species’ range [6,13] and rarely include populations at the extremes of their distribution. Understanding how foraging behaviour varies across the full extent of a species’ range could generate important insights into the causes and consequences of group living, as well as responses to global change.

Much research on foraging by colonial animals comes from seabirds. Over 95% of seabird species nest in colonies and they form some of the largest non-human vertebrate aggregations [14]. Density-dependent food competition arises in breeding seabirds where a zone of reduced prey accessibility occurs around colonies—Ashmole’s halo [3]. As a population grows, competition induces longer foraging trips, which reduces chick feeding rates and in turn reproductive success, as well as adult survival, eventually constraining colony size [3,15–18]. As well as direct measures of reduced prey availability around some colonies [5,19], support for this hypothesis comes primarily from inter-colony comparisons of foraging behaviour [6,8,9,13]. Density-dependent effects on seabird foraging interact with the effects of local environmental conditions [17]. This is important since reduced prey availability is thought to be the primary climate-related threat to seabirds [20], especially during breeding when birds are constrained to the colony [21,22]. Many seabird prey species are shifting poleward [23–26], and while some seabird populations mirror this [27–30], their ability to keep pace may be limited because individuals are generally faithful to their natal and breeding sites [31,32] and colony formation is rare [33].

Here, we study the foraging behaviour of Northern Gannets *Morus bassanus* (hereafter ‘gannet’) in relation to colony size and latitude. Gannet foraging trips are longer on average at larger colonies [6,17], and there is evidence for shorter foraging trips at more northerly colonies [34,35]. Gannets currently breed at 54 colonies across the North Atlantic, having experienced a rapid population increase over the last century [36,37]. This was probably influenced by reduced persecution [38] and increased food availability, potentially including fishery discards [39]. Since 1935, gannets have expanded their range northwards by 7.7°, with no southward change [27,40,41]. This may have been facilitated by ocean warming bringing influxes of prey in the north [42–45] but poor conditions in the south [46–49]. Increased availability of gannet prey species in the north, such as Atlantic Mackerel *Scomber scombrus* [42,44,45], may be linked to lower foraging effort [35]. More recently, population growth and range expansion may be impacted by thousands of gannets dying in an outbreak of highly pathogenic avian influenza (HPAI) H5N1 that began in 2021, but the scale of this is not yet fully known [50,51].

Here, we measured gannet foraging effort (foraging trip range and duration) from satellite tracking for 21 of the 54 known gannetries (varying in size from 138 to 60 953 nests and supporting approximately 73% of the global population [37]), spanning 89% of their latitudinal breeding extent. Birds were tracked for multiple years at some colonies, all before the recent HPAI outbreak. We tested how this covaries with colony size, latitude and year. We predict foraging effort to covary positively with colony size but also colonies at the expanding northern edge of the species’ range to have lower foraging effort than expected for their population size.

## 2. Methods

### 2.1. Foraging effort

**Figure 1.**
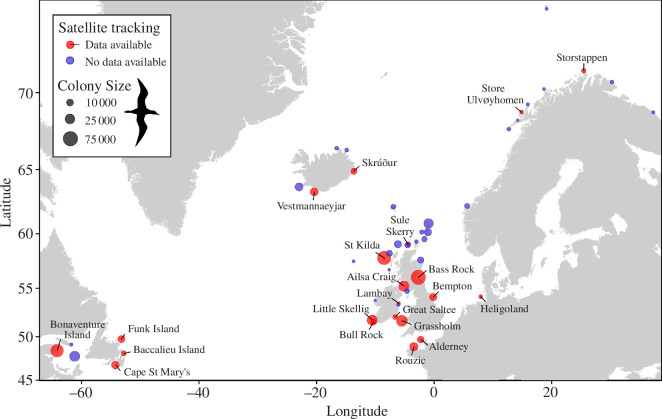
All 54 Northern Gannet *Morus bassanus* colonies with circles proportional to colony size. Red circles indicate 21 colonies for which satellite tracking data were used in this study. No data available for colonies indicated by blue circles. Map adapted from tiles by Stamen Design, under Creative Commons (CC BY 3.0) using data by OpenStreetMap, under the Open Database Licence.

We collated information on foraging trip duration and maximum distance travelled from the colony for 21 colonies (electronic supplementary material, figure S1), calculated from more than 7100 trips for 1118 chick-rearing gannets, collected between 1998 and 2021. Ranging from 46.81° N to 71.23° N (2714 km at equivalent longitude), colonies span 89% of the latitudinal extent of the species’ breeding colonies (3045 km at equivalent longitude) from Cape St Mary’s (46.81° N) to Funk Island (49.75° N) in Newfoundland, and from Rouzic (48.90° N) to Bjørnøya, Svalbard (74.21° N) in the Northeast Atlantic ([27,52]; figure 1).

We combined seven published means and 14 direct estimates from tracking data located via the BirdLife International Seabird Tracking Database accessed at www.seabirdtracking.org (figure 1, table 1, electronic supplementary material, table S1). Multiple years of tracking data [2–11] were available for 13 colonies. Where multi-year data were available, we used the mean across all trips to produce colony-level values unless otherwise indicated. Sampling frequency varied from 1 s to 180 min. Only Great Saltee had this lowest frequency, and only for five of the 71 birds tracked. Most movement data came from precision global positioning system (GPS) devices (archival and remote download), except for St Kilda, UK, where birds were fitted with platform terminal transmitters (PTTs; which relayed locations via the ARGOS satellite systems with a median of 75 min between locations [12]). PTTs are less likely to record at regular intervals compared with GPS loggers [69], so we removed foraging trips with poor data quality (one trip with only two records and 12 with location intervals over 3 h).

**Table 1.** Foraging effort and population size for 21 Northern Gannet *Morus bassanus* colonies ordered by latitude [12,35,47,48,53–64]. Values are given ± s.d. where available. See electronic supplementary material, table S1 for longitude, years of counts and tracking data, sampling frequency, sample size and total distance travelled. *Colonies in the West Atlantic.

colony	lat.	mean duration (h)	mean max. distance (km)	no. birds	source of tracking data	count (AOS/AON)	source of colony count
Cape St Mary’s*	46.81	14.3 ± 0.5	72.4 ± 1.9	22	[59]	14 598	[49]
Baccalieu Is*	48.15	9.3 ± 7.3	39.9 ± 24.7	6	[60]^a^	2253	[52]
Bonaventure*	48.48	28	132	14	[61]	53 635	
Rouzic	48.90	25.1 ± 10.9	124.2 ± 50.0	169	[48]	20 400	This study
Alderney	49.71	23.0	122.0	60	[47]	7885	[37]
Funk Is.*	49.75	16.5	102.6	26	[62]	10 047	[52]
Bull Rock	51.58	11.9 ± 8.1	69.8 ± 34.1	14	[12]^a^	3694	[65]
Grassholm	51.73	21.8 ± 1.7	116.8 ± 7.4	304	[63]	36 011	[66]
Little Skellig	51.78	13.4 ± 11.8	96.5 ± 61.8	9	[12]^a^	29 683	[65,67]
Great Saltee	52.11	21.5	98.5	71	[12]^a^; [64]; This study	4722	
Lambay	53.50	11.6 ± 7.6	37.5 ± 19.6	3	[12]^a^	138	JNCC 2010
Bempton	54.15	9.1	45.7	35	[53]; This study	11 061	JNCC 2012
Heligoland	54.18	7.9 ± 8.0	42.0 ± 45.7	3	[54]	656	[66]
Ailsa Craig	55.25	26.2 ± 16.2	152.3 ± 70.5	16	[12]^a^	33 226	
Bass Rock	56.08	25.6 ± 12.6	200.1 ± 98.5	206	[55–57]^a^	60 953	JNCC 2009
St Kilda	57.86	24.3 ± 14.8	164.2 ± 124.1	21	[12]^a^	60 290	[66]
Sule Skerry	59.08	14.4 ± 5.9	72.9 ± 19.8	2		1870	
Vestmann-Aeyjar	63.36	10.2 ± 7.4	43.0 ± 27.0	9	[35]	15 044	[68]
Skrúður	64.90	4.8 ± 4.5	29.2 ± 24.2	27		6051	
Store Ulvøyhomen	68.85	6.9	22.3	43	[58]	308	[27]
Storstappen	71.23	6.6	38.7	58		1244	

^a^ Mean values were not available in the published sources, so tracking datasets were provided by the authors.

AON, apparently occupied nests; AOS, apparently occupied sites; JNCC, Joint Nature Conservation Committee Seabird Monitoring Program database (http://jncc.defra.gov.uk/smp/ accessed 14 January 2019).

### 2.2. Colony size

We collated gannet colony size based on occupied sites or nests (range: 138−60 953) for the year closest to tracking studies, with a mean difference of 1.75 years (range: 0−7; table 1; electronic supplementary material, table S1). Our study includes data from 21 colonies with a combined estimated population of 382 906 nests, approximately 73% of the global population (525 694 pairs from 54 colonies [66]; table 1). We treated the islands of the Vestmannaeyjar archipelago including Hellisey, and Les Etacs and Ortac in Alderney, as single colonies because of their proximity (approx. 5 km).

### 2.3. Statistical analysis

We tested the effects of colony size and latitude on foraging trip duration and maximum distance using linear models. We square-root transformed colony counts as relationships between mean trip duration and square-root colony size are approximately linear [6] and because foraging area (km^2^) increases with the square of distance (km) [70]. Traditional standard error calculations assume infinite populations. This is not normally problematic when sampling from large populations, but when the sample is a large proportion of the total population (greater than 5–10%), the overestimation of the standard error becomes important [71,72]. As we sampled 21 of the 54 known colonies (39%), we implemented a finite population correction in the ‘survey’ R package’s ‘svyglm’ function [73], using a conservative upper estimate of 60 colonies worldwide. We used the Rao–Scott working likelihood test for model selection, using the default linear combination of *F* distributions with 17 denominator degrees of freedom to generate the likelihood ratio [74]. We calculated adjusted pseudo *r*^2^ values in the ‘jtools’ R package [75], and delta (Δ) pseudo *r*^2^ values for each explanatory variable to compare their relative importance. We also ran these models excluding the six colonies for which nine or fewer individuals were tracked (electronic supplementary material, table S2). We did not have a large enough sample size to test for an interaction between latitude and colony size. We also tested how well trip duration predicts maximum distance (Results in electronic supplementary material, figure S2).

As 13 colonies had multiple years (range: 2−10) of tracking data (electronic supplementary material, table S3), we separately fitted linear mixed models explaining annual mean trip duration and maximum distance in relation to colony size, and either latitude or longitude using the ‘spaMM’ R package [76]. We fitted year as a linear numeric variable to check for and control for any potential long-term trends over the 24-year study period (1998−2021). We included colony ID by fitting it as a random intercept. To account for any spatial autocorrelation, we applied a spatial random effect using a Matérn correlation function to the great circle distances between colonies [76]. We checked the residuals using simulation with the ‘DHARMa’ R package [77]. We did not apply a finite population correction to the standard error because we were not aware of an applicable method for linear mixed models with mean sample sizes of fewer than 10 years within each colony and fewer than 30 colonies [72]. R scripts and data are available at https://github.com/bethclark36/ForagingEffortGannets [78], archived at https://doi.org/10.5281/zenodo.12796423 [79].

## 3. Results

**Figure 2.**
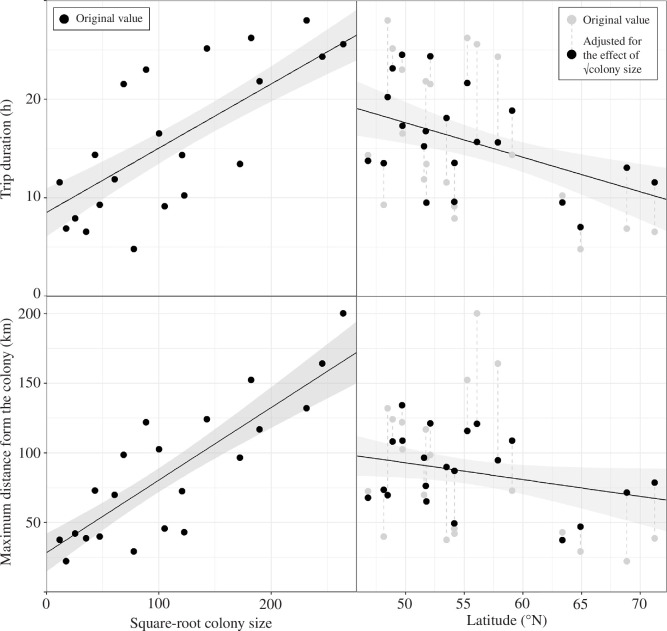
Mean foraging trip duration and maximum distance from the colony for 21 Northern Gannet *Morus bassanus* colonies in relation to colony size and latitude. Black lines show the prediction from a finite population corrected linear model ±95% confidence intervals (grey ribbon) using the mean value of latitude to predict the effect of square-root (√) colony size and vice versa. To visually correct for √colony size, we subtracted the √colony size effect (grey dashed line) from each data point (grey circle) and then added the √colony size effect for the mean √colony size (black circle). See electronic supplementary material, figure S3 for figure 2 with points labelled by colony name.

Foraging trip duration and maximum distance reached from the colony increased significantly linearly with square-root colony size, and trip duration significantly decreased with latitude—colony size had a much larger effect size than latitude (table 2; figure 2). Linear models including both square-root colony size and latitude had a pseudo-adjusted *r*^2^ of 0.62 for foraging trip duration and 0.72 for maximum distance. Results were similar when excluding six colonies with nine or fewer tracked individuals (electronic supplementary material, table S2).

**Table 2.** Parameter estimates for linear models fitted with the finite population correction explaining Northern Gannet *Morus bassanus* colony means for foraging trip duration and maximum distance for 21 colonies. Delta (Δ) pseudo-adjusted *r*^2^ is the difference in pseudo-adjusted *r*^2^ between the models with and without the explanatory variable included and are thus a measure of the contribution of each variable to explaining the variation in foraging effort.

foraging effort	explanatory variable	estimate ± s.e.	2log LR	d.f.	*p* value	Δ pseudo *r*^2^
trip duration (h) ~	intercept	27.808 ± 6.246	—	—	—	—
	√colony size	0.065 ± 0.007	78.15	1,18	<0.001	0.41
	latitude	−0.350 ± 0.101	11.99	1,18	0.003	0.08
maximum distance (km) ~	intercept	94.623 ± 37.554	—	—	—	—
	√colony size	0.521 ± 0.058	81.514	1,18	<0.001	0.62
	latitude	−1.202 ± 0.620	2.423	1,18	0.071	0.02

**Figure 3.**
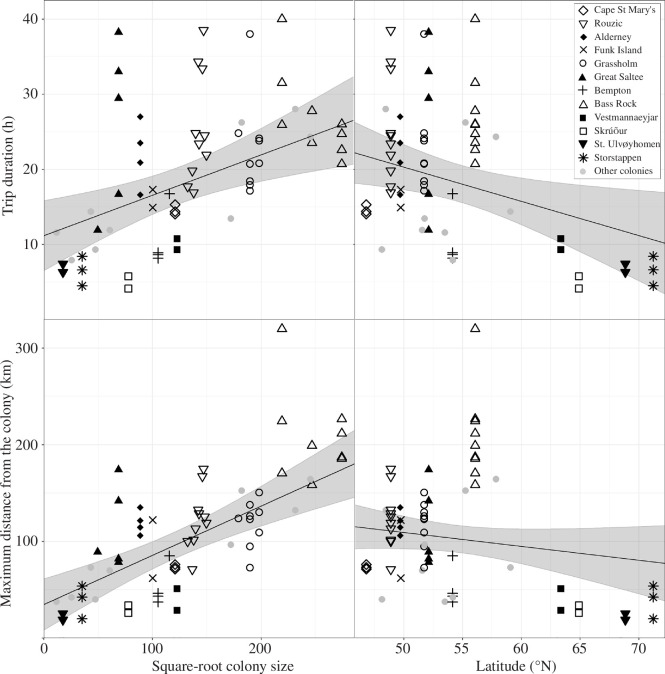
Annual mean foraging trip duration and maximum distance from the colony for 21 Northern Gannet *Morus bassanus* colonies in relation to colony size and latitude. Black symbols show the 13 colonies for which multiple years of tracking data were available (2−11 years). Grey circles indicate the eight colonies where only 1 year of data was available. Black lines show the prediction from a linear mixed model with colony fitted as a random intercept ±95% confidence intervals (grey ribbon) using the mean value of latitude to predict the effect of square-root colony size and vice versa.

When modelling annual means, trip duration and maximum distance increase with colony size and decreased with latitude (figure 3). Square-root colony size appeared in the top models for trip duration and maximum distance, with a substantial drop in pseudo *r*^2^ if not included (table 3). Latitude also appeared in the top and second-best models for trip duration, with the third model falling below the heuristic 2 ΔAIC cut-off. Longitude and the linear effects of year were consistently indicated to be of lesser importance than both colony size and latitude in these models, with their inclusion being limited to either models substantially worse than the top model, or in models with both colony size and latitude. The top models for maximum distance did not show a pattern of preferentially including latitude, longitude or the linear effect of year, indicating little evidence that they were having a large effect. Trip duration and maximum distance varied substantially at some colonies among years within the same colony (figure 3).

**Table 3.** Candidate models for linear mixed models for Northern Gannet *Morus bassanus* annual colony means for foraging trip duration and maximum distance from the colony, with colony and a distance matrix fitted as random effects. mAIC = marginal Akaike information criterion.

response	fixed effects				mAIC	Δ mAIC	pseudo *r*^2^
foraging trip duration ~	√colony size	+latitude			442.01	0	0.450
	√colony size	+latitude		+year	443.98	1.97	0.451
	√colony size				444.60	2.59	0.410
	√colony size		+longitude		446.47	4.46	0.411
	√colony size			+year	446.49	4.48	0.411
		latitude			447.87	5.86	0.379
	√colony size		+longitude	+year	448.35	6.34	0.412
		latitude		+year	449.30	7.29	0.384
			longitude		451.90	9.89	0.338
				year	452.47	10.46	0.332
			longitude	+year	452.97	10.96	0.348
	none				454.01	12.00	0.272
maximum distance from the colony ~	√colony size				655.24	0	0.632
	√colony size	+latitude			655.42	0.18	0.642
	√colony size		+longitude		656.41	1.17	0.636
	√colony size			+year	656.62	1.38	0.635
	√colony size	+latitude		+year	656.71	1.47	0.646
	√colony size		+longitude	+year	657.70	2.46	0.640
		latitude			668.74	13.50	0.545
		latitude		+year	670.64	15.40	0.546
			longitude		671.34	16.10	0.526
				year	671.78	16.54	0.523
	none				673.16	17.92	0.481
			longitude	+year	673.17	17.93	0.527

## 4. Discussion

Gannet foraging trip duration and maximum distance from the colony significantly increased with colony size, and after controlling for this, trip duration significantly decreased with latitude. We also recorded interannual variation within colonies but no long-term trend over the study period. We discuss these results in the context of density-dependent food competition, potential mechanisms of latitude effects, and how colonial animals might respond to global change.

### 4.1. Foraging effort and colony size

Our results provide a clear illustration that foraging effort increases with colony size in gannets, supporting previous studies of this species that used colony-based observations rather than biologging [6,17]. Our results are also consistent with multi-colony comparisons of other seabirds including shearwaters [80], tropical sulids [8] and guillemots [9]. Few studies have measured foraging behaviour across so many sites, but Patterson *et al*. [9] is a clear exception. They used tracking data from 29 murre colonies (7.6% of the 384 regional Common Murre *Uria aalge* and Thick-billed Murre *Uria lomvia* colonies) to estimate that foraging range scales with the cube root of colony size rather than the square-root used in our analysis.

Among gannets, relationships between colony size and foraging metrics were strong with delta pseudo *r*^2^ values of 0.41 for trip duration and 0.62 for maximum distance travelled. Differences in explanatory power between duration and distance may relate to individual foraging site fidelity. For example, adult gannets learn foraging locations [81], which are highly spatially repeatable both within and among years, while trip duration tends to be more variable presumably due to differences in routes, wind conditions and food availability among trips [56,82].

### 4.2. Interannual variation in foraging effort

Multi-year tracking revealed interannual variation in foraging effort within colonies, sometimes by tens of hours, but no detectable long-term trend over the study period. This is probably due to variable local environmental conditions since seabird foraging trips tend to be longer during years of poor environmental conditions [10,11,83,84]. ‘Good’ and ‘bad’ conditions for foraging gannets are unlikely to be synchronized across all colonies, especially for those that are far apart. Continued repeated tracking over a longer period could shed light on progressive changes in environmental conditions, particularly in a comparison between long-term trends in colonies in the far south and north.

### 4.3. Foraging effort and latitude

Controlling for colony size, we detected a latitudinal gradient in foraging trip duration and maximum distance. Such an effect was not detected in murres across a similar region [9]. There are at least three non-exclusive plausible explanations for why gannets have shorter foraging ranges in the north compared with similar-sized colonies further south. First, since gannets are primarily diurnal foragers, and generally rest on the water overnight [85], birds breeding in locations with long nights may have longer trips compared with high-latitude breeders experiencing shorter nights. Second, since many of the northerly colonies have fewer surrounding gannet populations, lower foraging effort may relate to reduced inter-colony competition as nearby colonies can increase intraspecific competition in seabirds [12,86–88]. Third, there may be an effect of climate change. At high latitudes, warming seas are associated with influxes of pelagic fish, potentially producing favourable gannet foraging conditions [27,42,44,89]. By contrast, warming at low latitudes is linked to reduced prey availability, high foraging effort and low breeding success on both sides of the Atlantic [46,47,59].

Understanding foraging ecology at high latitudes is timely because while gannet populations have increased throughout much of the twentieth and early twenty-first centuries [90], many seabirds on both sides of the North Atlantic have suffered declines in recent years, possibly linked to reduced food availability and quality driven by climate warming [65,91–93]. Most North Atlantic seabirds rely on forage fish, including sand eels *Ammodytes* spp. and Capelin *Mallotus villosus* [94], but warming reduces sand eel availability and quality [23]. For example, Atlantic Puffins *Fratercula arctica* breeding in declining populations had greater foraging ranges and lower chick provisioning rates [95]. Furthermore, Iceland and Norway have seen recent influxes of Atlantic Mackerel [42,44], which compete with and predate on sand eels and Capelin [96]. Similarly, increased Atlantic Herring *Clupea harengus* abundance has been linked to Capelin stock collapse and consequent decline in the vulnerable Black-legged Kittiwake *Rissa tridactyla* in Norway [89]. These changes heavily impact many seabird species, but mackerel and herring are excellent food for gannets due their large size and wide dietary breadth [39,45,97].

### 4.4. Mechanisms underpinning density dependence

The observed patterns are probably explained by density-dependent competition for food, leading to colony-specific halos of prey depletion or reduced prey accessibility [3]. Direct evidence for prey depletion by marine predators is scarce [5,19], but our results are consistent with previous evidence from comparing multiple colonies [6,8,9,13]. The strong positive relationship between population size and foraging trip length was apparent even though many of the sampled colonies are still growing [37] and therefore that food was not yet limiting colony size at some sites. Interannual variation in foraging effort indicates flexibility in response to fluctuating local environmental conditions, including marine heatwaves [49,98]. Breeding adult seabirds can somewhat buffer chick feeding against lower prey availability by increasing travel speed reducing resting time [98], but this may lead to carry-over effects and lower adult survival [18]. Our results are consistent with a reduced effect of competition at northern range margins for gannets [34,35]. This may allow for rapid population growth at small northern colonies and, therefore, a mechanism for a shift in the distribution of the species in response to climate change. However, we note that some northern colonies have decreased and some gone extinct, highlighting nuance in the patterns presented here [90]. We also suggest a more detailed analysis of the benefits as well as the costs of conspecific density since social interactions inform much about gannet foraging [99].

### 4.5. Monitoring and conservation

Monitoring foraging effort could allow us to identify struggling populations before demographic changes are detectable [18,95], particularly for long-lived, slow-breeding species [100]. Understanding the impact of environmental change on foraging behaviour may help mitigate climate change impacts by accounting for current and future foraging conditions in marine spatial planning [20], including for renewable energy development, and prioritizing restoration and area-based conservation measures [101,102]. Some species may be able to shift into more suitable regions, but some southward-shifting seabirds are already reaching the limits of available land for nesting [28,29]. Seabirds may also be restricted to foraging in suitable wave and wind regimes [103], and light availability near the poles [104,105]. Finally, the recent outbreak of HPAI (2021–2023; [51]) caused unprecedented seabird mortality across the North Atlantic and beyond, with thousands of gannets dying in 40 colonies on both sides of the Atlantic [50]. Our results provide baselines for gannet foraging trip metrics, which can be compared with data from during and after the outbreak to shed more light on density-dependence in colonial species. Five gannets tracked from the Bass Rock in 2022 made longer foraging trips during the outbreak and visited other colonies, which was not previously observed and could spread the disease [106]. After the 2022 outbreak, tracked gannets had shorter foraging trips than before, consistent with reduced intraspecific competition [106,107]. Reductions in colony size could greatly alter foraging seabird ranges with wide-ranging implications such as for marine spatial planning, further highlighting the importance of ongoing surveillance. Overall, understanding spatial variation in prey availability is key to future-proofing the conservation of seabirds and other colonial species.

## 5. Conclusion

Breeding gannets’ foraging effort increased with colony size across their latitudinal range, probably due to density-dependent competition for food. Moreover, we also found interannual variation, and an effect of latitude suggesting reduced intraspecific competition in the north of their distribution. These results, therefore, indicate how foraging flexibility may enable colonial animals to respond to environmental change but are ultimately limited by prey availability.

## Data Availability

R scripts and data are available at [78], archived at [79]. Gannet tracking data used in this study are available to request from the BirdLife International Seabird Tracking Database (https://www.seabirdtracking.org/) with the following dataset IDs: 716–725, 728–734, 955–956, 1341–1342, 1472–1473, 1475–1478, 1543, 1636, 1638, 1653, 1660, 1793–1796, 2201. Electronic supplementary material, table S1 contains additional data for the 21 gannet colonies [108]. Figure S1 shows the relationship between trip duration and maximum distance. Electronic supplementary material, table S2 gives model estimates for colonies for which more than 10 individuals were tracked. Electronic supplementary material, table S3 gives the values derived from GPS data and colonies counts separated by year.
